# Household catastrophic health expenditure and its effective factors: a case of Iran

**DOI:** 10.1186/s12962-021-00315-2

**Published:** 2021-09-16

**Authors:** Ramin Ravangard, Faride Sadat Jalali, Mohsen Bayati, Andrew J. Palmer, Abdosaleh Jafari, Peivand Bastani

**Affiliations:** 1 Health Human Resources Research Centre, School of Health Management and Information Sciences, Shiraz University of Medical Sciences, Shiraz, Iran; 2 Department of Health Services Management, School of Management and Medical Information Sciences, Shiraz University of Medical Sciences, Shiraz, Iran; 3 Student Research Committee, Shiraz University of Medical Sciences, Shiraz, Iran; 4 Menzies Institute for Medical Research, University of Tasmania, Hobart, TAS Australia; 5 Centre for Health Policy, School of Population and Global Health, University of Melbourne, Melbourne, VIC Australia

**Keywords:** Catastrophic Health Expenditures, Out-of-pocket payments, Financial contribution

## Abstract

**Background:**

The World Health Organization (WHO) has placed special emphasis on protecting households from health care expenditures. Many households face catastrophic health expenditures (CHEs) from a combination of economic poverty and financing the treatment of medical conditions. The present study aimed to measure the percentage of households facing catastrophic CHEs and the factors associated with the occurrence of CHEs in Shiraz, Iran in 2018.

**Methods:**

The present cross-sectional study was performed on 740 randomly selected households from different districts of Shiraz, Iran in 2018 using a multi-stage sampling method. Data were collected using the Persian version of the “WHO Global Health Survey” questionnaire. CHEs were defined as health expenditures exceeding 40% of households’ capacity to pay. Households living below the poverty line before paying for health services were excluded from the study. The associations between the households’ characteristics and facing CHEs were determined using the Chi-Square test as well as multiple logistic regression modeling in SPSS 23.0 at the significance level of 5%.

**Results:**

The results showed that 16.48% of studied households had faced CHEs. The higher odds of facing CHEs were observed in the households living in rented houses (OR = 3.14, P-value < 0.001), households with disabled members (OR = 27.98, P-value < 0.001), households with children under 5 years old (OR = 2.718, P-value = 0.02), and those without supplementary health insurance coverage (OR = 1.87, P-value = 0.01).

**Conclusion:**

CHEs may be reduced by increasing the use of supplementary health insurance coverage by individuals and households, increasing the support of the Social Security and the State Welfare Organizations for households with disabled members, developing programs such as the Integrated Child Care Programs, and setting home rental policies and housing policies for tenants.

**Supplementary Information:**

The online version contains supplementary material available at 10.1186/s12962-021-00315-2.

## Background

The World Health Organization in its 2000 report has declared that one of the goals of the health systems is a fair contribution to financing healthcare expenditures [1]. A fair and equitable system is one in which households contribute to their healthcare expenditures in proportion to their affordability and income [2]. The household’s financial contribution to the health system represents the financial burden imposed on the household by payments for health care [3]. Generally, part of all household incomes in societies around the world is spent on health care (ranging from $49 in Afghanistan to $10,623 in the United States in 2018) [4], the distribution and proportion of which may indicate the imposition of healthcare-related financial burdens on societies [1].

The rising costs of health services due to the development of technologies, and the increases in individuals’ health awareness and expectations have created problems in financing healthcare expenditure for people in the community [5]. Protecting people from the costs of diseases is one of the main goals of health systems [6]. Financial protection means that no household should spend more than a “reasonable proportion” of its income to finance special health services. This ratio, which includes both direct and indirect health expenditures, should not exceed the household’s ability to pay, which is the result of subtracting the minimum subsistence expenditure from the total household expenses over a given period. If healthcare expenditure exceeds this level, it can lead to catastrophic health expenditures and medical impoverishment [7, 8]. Wagstaff and Doorslaer [50] have proposed two basic approaches measuring catastrophic health expenditures (CHEs). The catastrophic health expenditure in the first approach is defined as follows: direct out-of-pocket payments for health care that go beyond a certain threshold of income or household expenses. In the second approach, poverty occurs if a household is driven below the poverty line due to direct out-of-pocket payments for health) [9]. According to the World Health Organization, a household is faced with CHEs when more than 40% of its ability to pay has been spent on healthcare expenditures [10].

According to the World Health Organisation 2008 report, about 100 million people worldwide are under the poverty line each year due to health care expenditures [10]. Studies conducted in different countries have shown that the percentage of households facing CHEs varies from country to country. For instance, the results of the studies conducted by Xu et al. [11, 12] in several countries showed that the percentage of households facing CHEs has varied from 1 to 15%. In other studies carried out in Brazil and Burkina Faso, the percentage of people facing catastrophic health care expenditures was 12% and 15%, respectively [13, 14]. Meemon et al. [15] reported that the rate of facing CHEs in 2019, after the implementation of the universal health coverage plan in Thailand was 2.78%, and in another study in 12 Latin American and Caribbean countries, the rates varied from 1 to 20% [16]. Similar studies have also been conducted in Iran. For example, in a systematic review, Doshmangir et al. [17] concluded that the rate of CHEs in Iran at the population level, across diseases, and among cancer patients was 4.7%, 24.3%, and 54.5%, respectively. In the study of Aryankhesal et al. (18), the overall rate of exposure to CHEs in Iran from 1984 to 2014 was reported to be 7.5% [18]. Moreover, in a study conducted by Ghiasvand et al. [19], the rates of facing catastrophic health care expenditures among rural and urban households in Iran varied from 0.5 to 14.3% and from 0.48 to 13.27%, respectively.

In general, it is important to review and monitor catastrophic expenditures in health systems and to identify factors that put households at risk of facing CHEs. This will help health policy policymakers choose preventive policies and corrective measures to address this problem [2]. Estimating the rate of facing the CHEs in societies is one indicator of the performance of health systems in health resource allocation and risk distribution [20]. In Iran, whose population is mainly covered by the health insurance system, measuring the impact of health care expenditures on households plays an important role in identifying groups and communities at risk and may allow the implementation of risk reduction policies in these groups [21]. It should be noted that health insurance coverage in Iran is provided by three main organizations, including Social Health Insurance (SHI) schemes, institutional health insurance funds, and commercial health insurance organizations. SHI schemes consist of three main insurance funds, including Iran Health Insurance Organization (which covers governmental employees and all individuals that were not eligible to be covered by other health insurance organizations, with the patient co-payment of 10% and 30% for inpatient and outpatient services, respectively), Social Security Organization (which is responsible for covering workers and employees in the private sector and the self-employed persons, and provides services in its health centres and hospitals free of charge for the SSO-insured people or with the 10% and 30% patient co-payment for respectively inpatient and outpatient services in other medical centres) [22, 23], and Armed Forces Medical Services Insurance Organization (which has been established by the Ministry of Defence, covers people working in the armed forces and their families, and provides services to the insured free of charge in the military medical centres or with a patient co-payment of 10% to 35% in private medical centres with which has a contract [24]. Institutional health insurance funds such as Petroleum Industry Health Organization, the National Broadcasting Organization, banks, etc. provide insurance services to their employees. And finally, commercial health insurance organisations such as Alborz, Mellat, Pasargadae, Atiyeh Sazan Hafez, etc. provide services that basic insurers do not provide, with a patient co-payment of 30% for outpatient and 10% for inpatient services [22, 23].

On the other hand, after the implementation of the targeted subsidy plan and the occurrence of severe inflation in Iran, which resulted in major changes in the financial situation of households [25], it was necessary to study the rate of CHEs and the associated factors in the whole country, as well as in different regions and provinces. Iranian studies such as those by Mohammadalizadeh Hanjani and Fazaeli [26], and Nekoeimoghadam et al. [27] reported rates of CHEs of 3.90% across the whole country, and 4.10% in Kerman. However, so far no similar study has been carried out in Shiraz city in Fars province. Therefore, this study aimed to measure the percentage of households facing CHEs and associated factors in Shiraz, Iran in 2018, hoping that its findings will provide information for the health authorities and policymakers of the country and the city of Shiraz to reduce the occurrence of CHEs.

## Methods

The present cross-sectional household survey was conducted in 2018. The study population included all households living in Shiraz, Iran. Shiraz is the fifth-most-populous city of Iran and the capital of Fars Province located in the southwest of Iran. The sample size was determined using the results of a pilot study through the use of the following formula, assuming α = 0.05, d = 0.04, p = 0.19, q = 0.81, and design effect = 2.

$$n = \frac{{Z_{{1 - \frac{\alpha }{2}}}^{2} pq}}{{d^{2} }}\quad n = \frac{{1.96^{2} \times 0.19 \times 0.81}}{{(0.04)^{2} }} = 370\quad 370 \times 2 = 740,$$

where n is the sample size, $Z_{{1 - \frac{\alpha }{2}}}^{2}$ is the critical value of the normal distribution at $1r\frac{\alpha }{2}$, p is the estimated proportion of household catastrophic health expenditure, q = 1 − p, and d is the desired level of precision.

The determined sample size was selected using the multi-stage sampling method through the stratified sampling method proportional to size, simple and systematic random sampling methods (Additional file 1:Sampling Methods).

The data were collected using the World Health organization’s questionnaire entitled “World Health survey”. It was developed in 2003 to measure the performance of health systems and was translated into Persian in the study of Kavousi et al. [28], in which its availability and reliability were confirmed. The questionnaire contained the following dimensions: household socioeconomic information, household expenditures (including food, housing, education, and all other expenditures), average monthly income, demographic characteristics of each household member, the presence of disabled family members, the total household costs for outpatient services (in the previous month), and the total household costs for inpatient services (in the last 12 months).

All data collected from Shiraz households were entered into Excel version 2007, and households were classified into quantiles based on their income and the number of households in each quintile. Health services expenditures at each quintile were calculated separately. The income and expenditures were presented based on the purchasing power parity (PPP) $ exchange rate of 22,075 Rials per $1 PPP according to the World Bank website [29].

In this study, the basis for estimating CHEs was the approach recommended by the World Health Organisation to investigating the fair financial contribution of households in the health system, which includes the calculation of the households facing CHEs and households driven below the poverty line as a result of health care consumption and health care expenditures (Additional file 2: Estimating CHEs) [12, 30–32].

It should be noted that households living below the poverty line before paying for health services were excluded from the study. The collected data were analyzed using SPSS 23.0 (SPSS Inc., Chicago, IL, USA), and the associations between the studied households’ characteristics and facing CHEs were determined using the Chi-Square test. Then, the simultaneous associations between all studied characteristics and facing CHEs were specified using a multiple logistic regression model. A P-value < 0.05 was considered statistically significant.

## Results

In the sample population, 51.50% were female, 57.60% were married, 35.76% were in the age group of 18–34, 45.60% had academic and university degrees, 22.7% were students, 95.00% had basic health insurance coverage (95%) especially Social Security Organization (61.18%), and 57.88% did not have any supplementary insurance coverage (Additional file 3: Demographic characteristics of the studied sample in 2018). Of the households studied, 122 households (16.48%) had encountered CHEs. The results of Chi-Square test showed that facing CHEs was associated with homeownership (P-value < 0.001), having disabled family members (P-value < 0.001), and having supplementary health insurance coverage (P-value < 0.001), so that out of 431 households living in the rented houses, 22% (93 households) faced CHEs. Also, Out of 213 households with a disabled person in the family, 44% (94 households) exposure to CHEs, and out of 338 households without supplementary insurance, 22% (76 households) faced CHEs (Table 1). It should be noted that these characteristics were associated with facing CHE, but the association here was unadjusted.

**Table 1:** Frequency of facing CHEs according to the studied household and individual’s characteristics

Characteristics	Facing catastrophic health expenditures				
	Yes N = 122 (16.49%)		No N = 618 (83.51%)		P-value
	Frequency	Percent	Frequency	Percent	
Household income quintile					
First	71	58.2	312	50.5	0.19
Second	32	26.2	222	35.9	
Third	8	6.6	48	7.8	
Fourth	7	5.7	20	3.2	
Fifth	4	3.3	16	2.6	
Having a family member over 65 years of age					
No	11	9	63	10.2	0.42
Yes	111	91	555	89.8	
Having children under 5 years old					
No	21	14.8	91	14.7	0.54
Yes	121	85.2	527	85.3	
Homeownership					
Tenant	93		338		< 0.001
Owner	29	23.8	280	453	
Having disabled family members					
No	28	23	499	78.3	< 0.001
Yes	94	77	119	21.7	
Having supplementary health insurance coverage					
No	76	62.3	262	42.4	< 0.001
Yes	46	37.7	356	57.6	
Household size					
1–2	18	14.8	120	19.4	0.75
3–4	83	68	352	57	
> 4	21	17.4	146	23.6	

*CHEs* catastrophic health expenditures

Multiple logistic regression showed that the probability of facing CHEs in households living in rented houses was 3.14 times more than homeowner ones (P-value < 0.001, CI 1.84–5.35), households with disabled members 27.98 times more than those without disabled members (P-value < 0.001, CI 6.12–32.79), households without supplementary health insurance coverage 1.87 times more than those with supplementary health insurance coverage (P-value = 0.01, CI 1.18–2.96), and households with children under 5 years old 2.72 times more than those without 5-year-old children (P-value = 0.02, CI 1.15–6.38) (Table 2).

**Table 2:** Estimates of the associations between studied households’ characteristics and facing CHEs using the multiple logistic regression model

Characteristics	Odds ratio	95% confidence interval (CI)	P-value
Income quintile			
First	1.09	0.35–3.38	0.87
Second	1.73	0.54–5.51	0.35
Third	1.50	0.39–5.65	0.55
Fourth	0.71	0.17–2.87	0.64
Fifth	1		
Homeownership			
Owner	1		
Tenant	3.14	1.84–5.35	< 0.001
Having disabled family members			
Yes	27.98	6.12–32.79	< 0.001
No	1		
Having supplementary health insurance coverage			
Yes	1		
No	1.87	1.18–2.96	0.01
Having a family member over 65 years of age			
Yes	1		
No	0.70	0.30–1.651	0.42
Having children under 5 years old			
Yes	2.72	1.15–6.38	0.02
No	1		
Household size			
1–2	0.93	0.47–1.83	0.84
3–4	0.76	0.41–1.41	0.38
> 4	1		

*CHEs* catastrophic health expenditures

## Discussion

Today, with the rising costs of health care, there is growing concern about the economic impact of health spending on households facing diseases [33], so that in most political circles and parties there is a discussion about health costs and protecting households from facing CHEs [32]. The present study aimed to measure the percentage of households facing CHEs and its effective factors in Shiraz, Iran in 2018.

Taking account of excluding households living below the poverty line before paying for health services from the study, the results showed that the percentage of households in Shiraz facing CHEs was 16.48%, which is higher than the goal of the Sixth Development Plan of the country, i.e. reducing the rate of facing CHEs to less than 1% [34]. This large gap can be due to the increasing costs of medical services, including pharmaceutical costs and costs of using new technologies, which put a lot of upward pressure on the health expenditure, and as a result, it places a heavy financial burden on households. Another important reason could be the financing of health care in developing countries largely through out-of-pocket payments, combined with the relative lack of adequate health insurance coverage [35]. Different rates of CHEs have been reported in previous studies in different health systems, both in Iran and other countries. For example, in a study by Rezaei et al. [36] in 2019, 4.12% of households in western Iran were exposed to CHEs. This rate was reported 11.80% by Kavousi et al. [28], 3.14% by Yazdi et al. [37], and 3.91% by Ghorbanian et al. [38] in the years 1995–2015 in Iran.

Also, in Zhen et al. in China [39], the percentage of households facing CHEs was 17.50% and in the study of Barasa et al. [40] in Kenya in East Africa was 6.58%.

The results of the multiple logistic regression in the current study showed that facing CHEs was significantly associated with living in rented houses, having disabled family members, not having supplementary health insurance coverage, and having children under 5 years old.

In other words, households living in rented houses had higher odds of facing CHEs (3.4 times) than those who owned a house, which is consistent with the results of the studies conducted by Mohammadzadeh et al. and Ghiasvand et al. [41, 42] in Iran. According to the Statistical Center of Iran, in 2018, 34% of the budget of urban households has been spent on renting a house, which has been the highest cost among household expenditures [43]. Therefore, the costs of renting a house and transporting home appliances when moving from house to house may impose an additional burden on household income, thereby lowering households' capacity to pay for healthcare.

However, Mobaraki et al. and Khammarnia et al. [2, 44] showed there were no significant associations between homeownership and facing CHEs.

In the present study, households with disabled family members were more likely to face CHEs (27.98 times). These households are more likely to be in need of care services and, consequently, because of the high costs of care services, they have fewer financial resources to meet other family needs. Also, such households have higher direct non-medical costs, including the costs of purchasing a wheelchair, changing the home environment to adapt to the situation of the disabled person, etc. Therefore, if there is no effective protection mechanism, these households face increased risks of financial problems and catastrophic expenditures. The results of the present study are similar to those of Hatam et al. and Kavosi et al.’s [28, 45] studies in Iran. Moreover, Somkotra and Lagrada, Gotsadze et al., and Su et al. [14, 46, 47] in their studies concluded that the presence of people with physical or mental disabilities in the household could increase the household health care costs over its total costs, increasing the risk of facing CHEs.

Furthermore, there was a significant association between supplementary health insurance coverage and facing CHEs in the current study, so that households that were not covered by supplementary health insurance had higher odds of facing CHEs (1.87 times) than those covered by supplementary health insurance. It can be due to that households covered by the supplementary health insurance schemes pay less to the health system, and the supplementary health insurance organizations provide services and cover costs that basic insurers do not provide and cover. In line with the results of the present study, Mobaraki et al. [44] demonstrated a significant negative association between supplementary insurance coverage and facing CHEs. Rezapour et al. [48] showed that having health insurance coverage could protect households from facing CHEs, similar to the results of the present study. Yardim et al. and Xu et al. [11, 49] showed that having basic and supplementary health insurance coverage had a positive effect on reducing the exposure to CHEs, which are consistent with the results of the present study. However, Mobaraki et al. [44] didn’t find any significant association between having basic and supplementary health insurance coverage and facing CHEs. On the other hand, the results of Wagstaff and Lindelow [50] in China showed that having health insurance coverage had increased the risk of households' exposure to CHEs by encouraging people to use more health services, especially more advanced services.

Moreover, the results of the current study showed that households with children under 5 years old were more likely to face CHEs. In other words, the higher the number of children under 5 years old in the household, the greater the risk of the financial burden on the household [51]. With the increase in the number of children under 5 years old, because of the greater need of this age group for health care and services as well as the high costs of childcare in healthy and suitable kindergarten and nursery school and buying food and dietary supplements needed by children, the households are more likely to face rising health costs and CHEs. These results are similar to those of the Mohammadzadeh et al., Sabermahani et al., and Amery et al.’s [4, 41, 52]. However, Hatam et al. and Kavosi et al. [28, 45] didn’t show any statistically significant association between having children under 5 years old and facing CHEs.

The results of the present study showed no significant associations between facing CHEs and household income and size as well as having a family member over 65 years of age, which are consistent with the results of Soofi et al. [53] and Kavousi et al.’s [28] studies, and inconsistent with those of the Mobaraki et al. [44], Emamgholipour et al. [54], Amery et al. [55], Ghiasvand et al. [42], Yardim et al. [49], Somkotra and Lagrada [56], and Su et al.’s [14] studies.

Overall, the reasons for the observed differences between the results of the current study and those of other studies mentioned can be due to the differences in the sources of data used, population and samples studied, sampling methods, data collection instruments and measuring tools used, how to determine the households’ total income (for example, asking the households or the use of expenditures as a proxy), and the year of study and therefore differences in the inflation rates and out-of-pocket payments.

Like other studies, the present study had some limitations such as recall bias, self-report and lower or higher cost reporting. Also, because this study was conducted only in one city of Iran, although the fifth-most-populous one, it is necessary to be cautious in generalizing the results of the present study to other Iran cities and provinces, as well as to other similar countries.

According to the result of the current study, policymakers should pay special attention to the poor and socioeconomically disadvantaged households to reduce their exposure to CHEs in Iran through measures such as making reforms in the basic health insurance service packages, supplementary insurance premiums, cost-sharing policies, and earmarked taxes allocated to the health system.

## Conclusion

According to the results of the present study, 16.48% of households in Shiraz suffered from CHEs. The results showed that households living in rented houses, having disabled family members, not having supplementary health insurance coverage, and having children under 5 years old were more likely to face CHEs. Findings indicate that the Iran health system has failed to meet the goal of the Sixth Development Plan of the country, i.e. reducing the rate of households’ exposure to CHEs to less than 1%. What is certain is that policymakers’ attention to the factors that increase the odds of facing CHEs can help reduce such expenditures and achieve the goal of financial protection for households. National health financing systems should design policies that not only allow people to access services when needed but also reduce the risk of people facing CHEs through reducing direct and out-of-pocket payments. In the long run, the goal should be to develop prepayment mechanisms such as social health insurance, tax-based financing, or combinations of prepayment mechanisms. It should be noted that in order to achieve such mechanisms, paying attention to the political, social, and economic context is one of the main priorities [57].

According to the results of the present study and in order to decrease the risk of facing CHEs and prevent many households from exacerbating their poverty or falling below the poverty line, the following suggestions can be offered: increasing the possibility of using supplementary health insurance coverage by individuals and households; increasing the support of the Social Security and the State Welfare Organizations for households with disabled members in order to cover all or part of the costs of treatment, medical equipment and nursing services they need, as well as paying pensions to the disabled or establishing special medical centres for taking care of the disabled requiring full care; developing programs such as the Integrated Child Care Programs to reduce the mortality and disability of children under 5 years of age, improve their growth and development, and reduce the financial burden of their diseases on households; setting home rental policies and housing policies for tenants; and reforming payment systems and supervising the approved tariffs for health services.

## Supplementary Information

**Additional file 1. **Sampling Methods.

**Additional file 2. **Estimating CHEs.

**Additional file 3.** Demographic characteristics of the studied sample in 2018.

## Data Availability

The datasets used and/or analyzed during the current study are available from the corresponding author on reasonable request.

## Abbreviations

CHEs: Catastrophic Health Expenditures
PPP: Purchasing power parity
OR: Odds ratio
