# Supplementary material for: Household catastrophic health expenditure and its effective factors: a case of Iran
Source: Cost Eff Resour Alloc. 2021 Sep 16;19:59. doi: 10.1186/s12962-021-00315-2 (PMC8444555; doi:10.1186/s12962-021-00315-2)
Supplement: Supplementary file 1 — Additional file 1. Sampling Methods. [file 12962_2021_315_MOESM1_ESM.docx]

**Sampling Methods:**

The determined sample size was selected using the multi-stage sampling method. In the first stage, Shiraz was geographically divided into 11 municipal districts, each of which was considered as one stratum. Within each stratum, the number of household samples was determined using the stratified sampling method proportional to size, based on the number of households in each stratum. In the second stage, after specifying the sample size of each stratum, the urban neighborhoods within the strata were considered as clusters. Subsequently, a neighborhood from each district was selected randomly for collecting data. In the third stage, the first house in the nearest alley on the right side of the southwest side of each neighborhood selected in the second phase was considered as the first household for sampling. Then, according to the number of households in each neighborhood and the sample size assigned to that district, and using the systematic random sampling method, the desired number of households were selected and sampled.
