# Supplementary material for: Household catastrophic health expenditure and its effective factors: a case of Iran
Source: Cost Eff Resour Alloc. 2021 Sep 16;19:59. doi: 10.1186/s12962-021-00315-2 (PMC8444555; doi:10.1186/s12962-021-00315-2)
Supplement: Supplementary file 2 — Additional file 2. Estimating CHEs. [file 12962_2021_315_MOESM2_ESM.docx]

**Estimating CHEs:**

In this study, the basis for estimating CHEs was the approach recommended by the World Health Organisation to investigating the fair financial contribution of households in the health system, which includes the calculation of the households facing CHEs and households driven below the poverty line as a result of health care consumption and health care expenditures. This calculation was carried out as follows:

Initially, household size and food expenditures were converted to equivalent household size and equivalent household per-capita food expenditures. In other words, in order to consider the economies of scale, instead of the household size, the equivalent household size was determined using the following formula [30]:

$${eqsize}_{h}={hhsize}_{h}^{\beta}$$

Where ${eqsize}_{h}= equivalent size$ , ${hhsize}_{h}= household size$, and $\beta=0.56.$

In order to obtain equivalent household per-capita food expenditures, the food expenditure of each household was divided by the equivalent household size. Then, for each household, the ratio of food expenditure to the total household expenditure was estimated, and next the households were ordered from the smallest to the largest ratio. The average food expenditure (the part of the total household expenditure spent on the food) of households in the 45th to 55th percentile was considered as the poverty line [31].

In the present study, it was equal to $ 5915.29 PPP. By considering this as the poverty line, the subsistence expenditure for each household was estimated separately using the following formula:

$SE={eqsize}_{h} \times PL$

In which SE = Subsistence Expenditure,${eqsize}_{h}= equivalent household size,$ and $PL=Poverty Line.$

To determine whether a household was above the poverty line, the subsistence expenditure was compared to the total expenditure of that household. If the total expenditure was less than the subsistence expenditure, the household was considered to be poor or below the poverty line [12].

The following formula was applied to estimate the capacity to pay for each household:

*CTP_i_ = EXP _i_ - SE_i_*

$$\mathrm{Where} \mathrm{CTPi}=Capacity to Pay, \mathrm{EXP}i=Expenditure , \mathrm{and} \mathrm{SE}i=Subsistence Expenditure.$$

In the above formula, if the household’s SE more than its food expenditure, SE would be replaced with the food expenditure.

Then, the ratio of each household's health expenditures to its capacity to pay was calculated. If the calculated number exceeded 40%, that household would be considered as a household facing CHEs.

The following steps were taken to identify households falling below the poverty line by paying for health services:

Each household's out-of-pocket payments were deducted from its total expenditures. If (EXP_i_) - (OOP_i_) < (SE_i_), the household had fallen below the poverty line [32].

In this formula, ${SE}_{i}=Subsistence Expenditure$*,* ${EXP}_{i}=\mathrm{Expenditure}$, and ${OOP}_{i}=Out of Pocket$ Payment.

Out-of-pocket payments included direct payments made by the households at the time of receiving health services.
