# Supplementary material for: Household catastrophic health expenditure and its effective factors: a case of Iran
Source: Cost Eff Resour Alloc. 2021 Sep 16;19:59. doi: 10.1186/s12962-021-00315-2 (PMC8444555; doi:10.1186/s12962-021-00315-2)
Supplement: Supplementary file 3 — Additional file 3. Demographic characteristics of the studied sample in 2018. [file 12962_2021_315_MOESM3_ESM.docx]

**Demographic characteristics of the studied sample in 2018**

**(Number of people in 740 households participating in the study: 2357)**

| Characteristics | | Frequency (%) |
| --- | --- | --- |
| Sex | Female | 1214 (51.50) |
|  | Male | 1143 (48.50) |
| Marital Status | Single | 1001 (42.40) |
|  | Married | 1356 (57.60) |
| Age (years) | <17 | 513 (21.76) |
|  | 34 – 18 | 843 (35.76) |
|  | 51 – 35 | 641 (27.19) |
|  | 68 – 52 | 295 (12.51) |
|  | > 69 | 65 (2.76) |
| Education | Illiterate^[[1]](#footnote-1)^ | 192 (8.10) |
|  | Elementary School | 338 (14.30) |
|  | Secondary School | 220(9.40) |
|  | High School | 532 (22.60) |
|  | Academic and University | 1075 (45.60) |
| Employment Statues | Unemployed | 279 (11.80) |
|  | Self-employed | 316 (13.35) |
|  | Working in the public sector | 258 (11.00) |
|  | Working in the private sector | 446 (19.00) |
|  | Student | 535 (22.70) |
|  | Housewife | 386 (16.35) |
|  | Retired | 137 (5.8) |
| Having Basic Insurance Coverage | Yes | 2239(95.00) |
|  | No | 118 (5.00) |
| Types of Basic Insurance | Social Security | 1442 (61.18) |
|  | Iran Health Insurance | 431 (18.29) |
|  | Imam Khomeini Relief Foundation | 13 (0.55) |
|  | Armed Forces | 94 (3.99) |
|  | Others | 259 (10.99) |
|  | Don’t have | 118 (5.00) |
| Having Supplementary Insurance Coverage | Yes | 993 (42.12) |
|  | No | 1364 (57.88) |

1. . Includes everyone in the community, including children under school age [↑](#footnote-ref-1)
